# Lymphatic drainage of cerebrospinal fluid in mammals – are arachnoid granulations the main route of cerebrospinal fluid outflow?

**DOI:** 10.2478/s11756-018-0074-x

**Published:** 2018-06-27

**Authors:** Wojciech Sokołowski, Karolina Barszcz, Marta Kupczyńska, Norbert Czubaj, Michał Skibniewski, Halina Purzyc

**Affiliations:** 1 Department of Morphological Sciences, Faculty of Veterinary Medicine, Warsaw University of Life Sciences – SGGW, Nowoursynowska 159, 02-776 Warsaw, Poland; 2 Faculty of Veterinary Medicine, Department of Surgery, Wroclaw University of Environmental and Life Sciences, Pl. Grunwaldzki 51, 50-366 Wroclaw, Poland

**Keywords:** Cerebrospinal fluid, Lymphatic drainage, Cribriform plate, Hydrocephalus

## Abstract

The outflow of the cerebrospinal fluid (CSF) in animals was over the years the subject of detailed analysis. For a long time it was stated that arachnoid granulations of the venous sinuses play a key role in CSF circulation. However, recent studies on this subject have shown that a considerable part of the CSF is drained to the lymphatic vessels. Moreover, disorders in the CSF passage may result in severe central nervous system diseases such as e.g. hydrocephalus. In this paper, we summarize the current knowledge concerning the lymphatic drainage of the CSF in mammals. We present in detail comparative anatomy of different species taking into account cranial and spinal compartment. In addition, we clarified role of the lymphatic vessels in the CSF outflow and the relationship between impairment in this transport and central nervous system diseases. In the author’s opinion knowledge on CSF circulation is still poorly examined and therefore required comment.

## Introduction

Until recently, arachnoid granulations protruding into dural venous sinuses were seen as the main outflow tract of cerebrospinal fluid (CSF) from the subarachnoid space (SAS). However, some authors reported other CSF outflow tracts as far back as in nineteenth century (Schwalbe 1869). For a long time these theories were marginalized but lately they gained importance. It is now accepted that a considerable amount of CSF passes from the SAS into lymphatic vessels (Bradbury and Cole 1980; Boulton et al. 1998, 1999; Mollanji et al. 2001). There is also an increasing number of reports stating that the impairment of the lymphatic outflow may result in central nervous system diseases such as hydrocephalus (Nagra et al. 2008). The aim of this paper is to systematize the current knowledge of the structure and functions of the lymphatic outflow tracts of cerebrospinal fluid while taking into account the diseases resulting from blocking those tracts.

## The structure of the connections between the subarachnoid space and the lymphatic system

### Cranial compartment

It is widely accepted that nerve tissue has no lymphatic vessels. At the same time, there is ample evidence that dyes and markers introduced (both in vivo and postmortem) intraventriculary or directly into the SAS enter the lumen of lymphatic vessels (Bradbury and Cole 1980; Erlich et al. 1986; Kida et al. 1993; Miura et al. 1998; Killer et al. 1999; Johnston et al. 2004; Zakharov et al. 2004; Lüdemann et al. 2005). CSF outflow tracts into the lymphatic system have been studied in mice, rat, rabbit, cat, dog, pig, sheep, monkey and humans (Bradbury and Cole 1980; Erlich et al. 1986; Kida et al. 1993; Löwhagen et al. 1994; Hunter et al. 1995; Brinker et al. 1997; Miura et al. 1998; Killer et al. 1999; Johnston et al. 2004; Zakharov et al. 2004; Lüdemann et al. 2005; Mathieu et al. 2013). It is known that CSF leaves the SAS with some of the cranial nerves (Evans and de Lahunta 2013). CSF accompanies the olfactory nerves and flows from the SAS, through the foramina of cribriform plate, into the nasal cavity where it fills lumen of the lymphatic vessels. This was confirmed by the postmortem studies which used the Microfill, silicon injection rubber compound. Microfill was introduced under pressure to the cerebellomedullary cistern and filled small lymphatic vessels located in the submucosal membrane of the ethmoid turbinal systems and the nasal septum (Mollanji et al. 2001; Zakharov et al. 2003, 2004; Johnston et al. 2004). In euthanized dogs, a solution of barium sulphate administered by the same route also reached the nasal cavity. Its presence was confirmed in X-ray images (Potts et al. 1972).

A direct connection between the cerebral SAS and the lymphatic vessels of the nasal cavity was revealed in microscopic studies of rabbits. Erlich et al. (1986) reported that in the cribriform plate area, the dura surrounding the olfactory bulbs joined the periosteum. Olfactory nerves, in their further course in nasal cavity, were surrounded only by the extension of the arachnoid connected with the cells of the perineurium. There were no blind endings of the SAS revealed. Its direct extension in nasal cavity was the perineural space comprised between perineurium and the olfactory nerves. Moreover, the perineural space freely combined with the spaces free of the connective tissue in the nasal mucous membrane, the construction of which resembled lymphatic vessels. These observations were confirmed by intraventricular administration of ferritin at intravital conditions. Its presence was noted in the perineural space, between the cells of the perineurium and in the above mentioned open spaces located in the connective tissue. According to the authors, the histological structure of the tissues in this area may suggest that the outflow of CSF is dependent on the difference of pressures between the SAS and the extracellular space of the nasal mucous membrane. Higher pressure of CSF in the SAS was supposed to open the perineural space and allow the outflow of CSF into the nasal cavity, as well as an increase in pressure in the nasal mucous membrane would squeeze the perineural space leading to its closure.

Histological studies of the cribriform plate area in rat also showed the presence of a direct connection between the SAS and the nasal cavity. During the study, india ink was administered into the cerebellomedullary cistern of the living animals. Then, in the appropriate time intervals, the animals were euthanized and the india ink location was determined. In the cribriform plate area, the researchers reported some winding channels transporting the dye directly from the SAS into the area of the nasal mucous membrane, where it reached the lymphatic vessels. These channels were mostly formed along the fila olfactoria. India ink was also located in the perineural space, but in this species no connection was observed between this space and the mucous membrane. Different observations may be due to differences between species or the use of different markers and their distinct properties (Kida et al. 1993).

The microscopic studies in humans with the use of India ink did not show a direct connection between the perineural space of the olfactory nerves and the lymphatic vessels of the nasal cavity (Löwhagen et al. 1994). These results contradict the observations conducted in humans during studies using Microfill injected into the cerebellomedullary cistern. It was visualized macroscopically that Microfill filled the perineural space, the lymphatic vessels of the nasal cavity and the direct connections between these two spaces (Johnston et al. 2004).

Another, although of lesser significance, CSF outflow tract is the area of the optic nerve. Unlike other cranial nerves, the optic nerve is a white matter pathway surrounded by the SAS filled with CSF (Killer et al. 2007). Therefore, contrast agents introduced into the cerebellomedullary cistern also surround the optic nerve (Potts et al. 1972; Brinker et al. 1997). It is known that these agents are able to leave the SAS of the optic nerve and pass to the soft tissues of the orbit (Brinker et al. 1997). Killer et al. (1999) studied the human optic nerve together with its surrounding meninges. In its bulbar part a dense network of lymphatic vessels located in the inner part of the dura were described. The particles of india ink introduced into the SAS of the optic nerve were described in the extracellular spaces of the dura, in the area of the lymphatic vessels and in their lumen. Structural features of the arachnoid cells of this area also indicated the existence of a direct connection between the SAS of the optic nerve and the lumen of the lymphatic vessels. It corresponds to the observations by Brinker et al. (1997), who noted a leakage of the contrast agent in rat from the SAS of optic nerve to the surrounding tissues where the nerve gets into contact with the sclera. Lüdemann et al. (2005) provided similar observations in cat finding the contrast agent in lymphatic vessels of the conjunctivae. Gomez et al. (1985) found no evidence in rabbits for a connection between the SAS of the optic nerve and the lymphatic vessels.

To a small degree, CSF is also transported to the structures located within the petrous part of the temporal bone. CSF passes to the perilymph of the inner ear via the perilymphatic duct located in the cochlear canaliculus, which connects the SAS of the cranial cavity with the scala tympani of the cochlea (Brinker et al. 1997; Evans and de Lahunta 2013). It is then absorbed into the lymphatic vessels of the tympanum (Arnold et al. 1971).

The presence of lymphatic vessels, which play a role in the absorption of CSF, were also described in the dura. Kida et al. (1993) demonstrated that lymphatic vessels of the dura in the area of the frontal lobes get filled with india ink following its administration to the cerebellomedullary cistern. The lymphatic vessels were also observed in the dura, in the area of the jugular foramina, close to the venous sinuses (Földi et al. 1966; Andres et al. 1987). In dog, lymphatic vessels in this area are located along cranial nerves (Földi et al. 1966).

The drainage basin for the lymphatic vessels participating in the CSF outflow from the cranial cavity are lymph nodes of the head and neck. Some links were demonstrated between the lymphatic vessels participating in the CSF outflow and mandibular lymph nodes in mice and dog, retropharyngeal lymph nodes in sheep, dog and cat, as well as cervical lymph nodes in mice and rat (Hasuo et al. 1983; Love and Leslie 1984; Kida et al. 1993; Hunter et al. 1995; Boulton et al. 1999; Johnston et al. 2004; Mathieu et al. 2013).

### Spinal compartment

The cranial cavity is not the only area from where CSF is drained by the lymphatic vessels. Lymphatic vessels are also involved in the absorption of CSF from the SAS area of the spinal cord. Miura et al. (1998) proved that in Japanese monkey (*Macaca fuscata* Blyth, 1875) a rich network of lymphatic vessels that drained CSF to the nearby lymph nodes originated in the meninges of the spinal cord, at the level of the cervical and the first thoracic segments. This network was mainly located in the epidural connective tissue surrounding area of the spinal nerve roots and discharged its contents into the deep cervical and prevertebral lymph nodes. In the remaining part of the thoracic region and the lumbar and sacral regions, in the area of spinal nerve roots there were only a few lymphatic vessels observed. The existence of the connection between the SAS of the spinal cord, and the lymphatic system were also found in mice. In this species, ^125^I-Human Serum Albumin administered intraventricularily reached the lumbar lymph nodes in large quantities (Boulton et al. 1999).

The information mentioned above describes a wide network of connections between the SAS and the lumen of lymphatic vessels. On this basis it might be concluded that the lymphatic system plays an important role in CSF absorption.

## The role of the lymphatic vessels in CSF absorption

According to the literature there are considerable differences between species in the lymphatic vessels role in the CSF drainage from the cranial SAS. Various studies with isotopically labeled albumin administered intraventriculary found that at least 50% of CSF in rat, 30% in rabbit, 10–15% in cat and 40–48% in sheep was transported to the lymphatic vessels (Bradbury and Cole 1980; Boulton et al. 1998, 1999). In another study carried out by Mollanji et al. (2001), it was demonstrated that in sheep almost 100% of CSF reached lymphatic vessels under low intracranial pressure. The obtained data clearly indicate that the lymphatic system plays an important role in the outflow of CSF from the area of the cranial cavity in the examined species of mammals.

The discrepancies in the results described above, may be due to differences between species, as well as to the use of different research methodologies. Mollanji et al. (2001) observed the change in the intracranial pressure in two groups of sheep: with damaged and preserved CSF outflow tract to lymphatic vessels through the cribriform plate. It was found that the participation of the lymphatic system in CSF outflow varied depending on the value of the intracranial pressure. In conditions of low intracranial pressure, almost 100% of CSF drained from the cranial cavity to the lymphatic vessels through the cribriform plate in the vicinity of the olfactory nerve. Blocking this route caused an increase of intracranial pressure and CSF outflowing with alternative tracts such as arachnoid granulations and lymphatic vessels associated with other cranial nerves. According to the authors, the results obtained in previous research on sheep (40–48% CSF outflow into the cervical lymph vessels) could be underestimated due to potential albumin “leakage”. The quantity of albumin introduced intraventriculary was measured in the lymph collected from the major cervical lymphatic vessels with previous closure of other lymphatic vessels in that area. Closing the lumen of all small vessels, which may be the pathway of a potential “escape” of albumin, is virtually impossible. It could affect the undervaluation of the obtained percentage data. The lymphatic vessels were ligated in a similar way also in the studies on rat, the results of which were quoted above. In experiments conducted on rabbit and cat cervical lymphatic vessels were not ligated at all (Bradbury and Cole 1980; Boulton et al. 1999). The use of those different study methods does not allow to specify the percentage participation of lymphatic vessels in CSF outflow from the cranial SAS in the studied species. The role of the lymphatic system in this process may be underestimated.

The quoted studies included a detailed description of lymphatic system involvement in CSF outflow and contested the dominant role of arachnoid granulations in some species. However, it did not specify what percentage of the CSF drained through arachnoid granulations and when this route gained importance. Zakharov et al. (2004) continued their studies on sheep using ^131^HSA injected into the cerebellomedullary cistern. The authors compared radioactivity of blood samples collected from the cranial venous system and the peripheral blood under different intracranial pressure states. The authors found that under normal intracranial pressure conditions, the main tract of CSF outflow was through the lymphatic vessels. The transportation to the cranial venous system, with the participation of arachnoid granulations, was triggered only when this pressure raised. In conditions of high intracranial pressure, the flow of lymph through the lymphatic cervical vessels also increased, which clearly indicated an increase in CSF transportation (Hasuo et al. 1983; Love and Leslie 1984).

Studies on rat confirmed that the lymphatic system plays the main role in CSF outflow from the cranial cavity. In this species, [^14^C] sucrose administered intraventriculary did not reach the SAS located over the cerebral cortex, thus it did not reach arachnoid granulations protruding into the sagittal dorsal sinus (Ghersi-Egea et al. 1996). Therefore, since CSF does not reach the area of arachnoid granulations, it can be concluded that it may be mostly drained outside the cranial cavity, in the vicinity of cranial nerves, and then it gets to lymphatic vessels. The results obtained by Murtha et al. (2014) seem to confirm this theory. The contrast agent administered intraventriculary did not appear in the venous sinuses, but it was largely drained through the cribriform plate in proximity to the olfactory nerve. Rats which received injections of kaolin into the basal cisterns, proved to have impairment of CSF outflow into the nasal cavity through the cribriform plate and the enlargement of the elements of the ventricular system. It is worth noting that the largest enlargement of the cranial ventricles occurred in those individuals whose CSF transportation through the cribriform plate was limited to the greatest extent (Nagra et al. 2008).

The cranial cavity is not the only compartment where CSF is absorbed from the SAS. A significant role in this respect is also played by the spinal compartment. In meninges of the spinal cord, in the vicinity of the roots of the spinal nerves, the presence of both the arachnoid granulations and the lymphatic vessels was identified (Kido et al. 1976; Miura et al. 1998). In studies conducted on sheep and cat it was stated that, respectively, about 25 and 16% of the total CSF produced was discharged by means of perispinal outflow tracts (Marmarou et al. 1975; Bozanovic-Sosic et al. 2001). Also in rat the spinal outflow tracts play an important role (Boulton et al. 1999). In studies on monkey it was reported that some part of the CSF was transported to the lymphatic vessels. The living animals received nanoparticles of carbon to the cerebellomedullary cistern, which was later found during autopsy in the lymphatic vessels of spinal meninges and surrounding lymph nodes (Miura et al. 1998). Potts et al. (1972) found the presence of contrast agent in dog, in the lumen of the intercostal and lumbar veins though the agent was administered to the SAS of the spinal cord. These observations may indicate that arachnoid granulations exists, however it has to be noted that the experiment was performed on corpses and this effect was achieved when the pressure difference between SAS and the lumen of venous vessels significantly exceeded the intravital conditions. The importance of the lymphatic and blood systems in CSF absorption still remains little understood and requires further research.

## Dynamics of CSF outflow through lymphatic vessels

CSF transportation from the cerebellomedullary cistern to the lymphatic vessels occurs rather quickly. In studies carried out intravitally on rat, Brinker et al. (1997) demonstrated that the contrast agent deposited to the cerebellomedullary cistern flowed from the caudal fossa, through the basal cisterns into the cribriform plate where it rapidly penetrated into the nasal cavity. The outflow of the contrast agent through the cribriform plate took place before the absorption through such tracts as the distal portion of the optic nerve and the structure of the inner ear and started in just 7 min from its administration. Some penetration of the contrast agent into venous sinuses was also observed. However, it should be noted that the experiment was carried out under conditions of increased intracranial pressure. Murtha et al. (2014) injected a contrast agent into the lateral ventricles of the brain of rat. Researchers demonstrated differences in the transportation rate of the contrast agent between different age groups. The contrast agent was transported successively through the third ventricle, mesencephalic aqueduct, the fourth ventricle, SAS in proximity to the skull base, from where it reached the area of the cribriform plate. This transportation was faster in older animals. The contrast agent reached the area of the cribriform plate after 21 min in older animals and in 23.8 min in younger individuals (the presented values are the median, and the flow time was significantly different between individuals). In this case, no transportation to the venous sinuses was noted, and a substantial amount of the contrast agent got into the SAS of the spinal cord.

## Impaired CSF outflow through the cribriform plate and CNS diseases

Several research teams reported that in rat CSF transportation to the cribriform plate took place in SAS in proximity and along to the base of skull (Kida et al. 1993; Ghersi-Egea et al. 1996; Brinker et al. 1997). After the intravital administration of India ink to the cerebellomedullary cistern, Kida et al. (1993) observed that it laid along the base of the skull and accumulated in large amounts in the basal cisterns, cisterns located in the area of hypothalamus and cerebral arterial circle. Rostrally, the dye flowed along the olfactory bulbs and reached the cribriform plate.

Similar conclusions might be drawn from other studies on rat, in which kaolin was deposited in basal cisterns. In those animals, a significant impairment of CSF outflow through the cribriform plate into the nasal cavity was obtained. It is worth noting that kaolin did not exceed rostrally the level of the optic chiasm and it did not contact with the cribiform plate in the examined specimens. Moreover, the studied animals developed communicating hydrocephalus, the degree of which was related to the degree of impairment of CSF outflow into the nasal cavity (Nagra et al. 2008). This suggests that impeding CSF outflow through lymphatic vessels has a significant impact on CSF drainage from the SAS (Nagra et al. 2010).

Other research that are worth considering are those carried out on homozygous mice *Pdn/Pdn* (Naruse and Ueta 2002). They are characterized by a set of defects, which include polydactyly, hydrocephalus and arhincephally, i.e. lack of olfactory bulbs (Keino et al. 1994). The study proved that in the investigated subjects, the dorsal sagittal sinus was narrow, and the arachnoid granulations were difficult to observe, which could be the cause of the resulting hydrocephalus (Naruse and Ueta 2002). However, a simultaneous lack of olfactory bulbs and possible impairment of CSF outflow to the lymphatic vessels of the nasal cavity should be considered.

Duong et al. (2000), analyzed more than 250 cases of people who had undergone surgery of the skull base. In 8% of them postoperative hydrocephalus developed though prior to the treatment none of the patients showed any symptoms of this disease. Haemorrhage and scarring in this area may impair CSF circulation and perhaps are a factor responsible for the formation of hydrocephalus.

Impaired lymph drainage from the area of head may also be responsible for the brain edema. Casley-Smith et al. (1978) caused chronic cervical lymphostasis, by ligation of lymphatic vessels of the neck and removal of cervical lymph nodes in cats. The lock of the drain lasted about 8 months, after which the animals were subjected to autopsy. In all studied specimens, brain edema and flattening of gyri and sulci occurred. Similar observations were also carried out in rabbits and cats, in which the lymph drainage blockade lasted three weeks (Casley-Smith et al. 1976).

The effects of abnormal CSF outflow through lymphatic vessels do not appear only in the chronic cases. Mollanji et al. (2001) demonstrated that cribriform plate obstruction in sheep resulted in an increase of intracranial pressure compared to the control group. The values of intracranial pressure increased in animals suffering from cribriform plate obstruction for 3–5 h after the surgery, and after that time, they reached a new, constant level, usually exceeding twice the value before the surgery. It should be emphasized that in all animals involved in the studies, laminectomy of C1-C2 with ligation of meninges was performed. It closed the connection between the cranial compartment and the spinal compartment, and thus cut off some compensatory mechanisms that might remove CSF excess.

## Conclusions

Despite differences between species, the information presented in this article clearly prove that in mammals, lymphatic vessels, and mainly lymphatic vessels of the nasal cavity, play a very important role in CSF absorption from the area of SAS. It seems that it is necessary to harmonize the methodology of research in this field, which would allow for an objective assessment of interspecies differences in terms of the percentage of the lymphatic system in the CSF drainage. Further studies would provide a better understanding of CSF absorption and, perhaps, would allow to determine the etiology and effective treatment of certain CNS diseases, for instance hydrocephalus.
